# Deadbug Bridging Performance in 6- to 15-Year-Old Competitive Alpine Skiers—A Cross-Sectional Study

**DOI:** 10.3390/biology11020329

**Published:** 2022-02-18

**Authors:** Gerda Strutzenberger, Lynn Ellenberger, Björn Bruhin, Walter O. Frey, Johannes Scherr, Jörg Spörri

**Affiliations:** 1 Sports Medical Research Group, Department of Orthopedics, Balgrist University Hospital, University of Zurich, 8008 Zurich, Switzerland; gerda.strutzenberger@balgrist.ch (G.S.); le@hest.ethz.ch (L.E.); walterofrey@hin.ch (W.O.F.); johannes.scherr@balgrist.ch (J.S.); 2 University Centre for Prevention and Sports Medicine, Department of Orthopedics, Balgrist University Hospital, University of Zurich, 8008 Zurich, Switzerland; 3 Motion Analysis Zurich, Department of Orthopedics, Balgrist University Hospital, Children’s Hospital, University of Zurich, 8008 Zurich, Switzerland; 4 Swiss-Ski, 3074 Muri bei Bern, Switzerland; bjoern.bruhin@swiss-ski.ch

**Keywords:** skiing, athletes, biological maturation, physical conditioning, injury prevention, physical fitness, core stability, testing

## Abstract

**Simple Summary:**

In competitive alpine skiing, a stable trunk plays an important role in increasing performance and preventing injuries. The aim of the present study was to investigate the ability to stabilize the trunk during so-called deadbug bridging (DBB) exercises, a stabilizing, dynamic movement task in the supine position with the pelvis lifted and unilateral leg raising, in 6- to 15-year-old skiers. Trunk stabilization performance was better in female skiers of the under 15-year (U15) category than in their male counterparts, whereas there was no sex difference at ages under 10 years (U10). The only potential confounder when testing youth skiers revealed being body height, but only in female U10 skiers. In summary, this study provided sport-specific normative reference data that may be of equal interest to both researchers and sport practitioners.

**Abstract:**

In competitive alpine skiing, a superior antirotation and rear-chain stabilization capacity is essential to constantly remain in dynamic equilibrium while skiing and to counteract the ski-specific adverse loading patterns of the back. As such, skiers’ trunk stabilization performance during deadbug bridging (DBB) exercises has been shown to be associated with both skiing performance and overuse complaints of the lower back in skiers under 16 years of age (U16). However, to date, little is known about the corresponding stabilization abilities in younger skiers, i.e., 6- to 15-year-old skiers. As part of a biomechanical field experiment during a national off-snow fitness competition, a total of 101 youth competitive alpine skiers were tested with respect to their trunk stabilization performance during DDB exercise. The maximum contralateral displacement of the pelvic drop during leg lift (DBB_displacement_) was quantified using reflective markers and a motion capture system (Vicon, Oxford, UK). Potential age group and sex differences in DBB_displacement_ were assessed using analysis of variance (ANOVA) at *p* < 0.05. Within each subgroup, the associations of DBB_displacement_ with age, anthropometrics and maturity offset were analysed using Pearson’s correlation (*p* < 0.05). Female skiers under 15 years of age (U15) showed better DBB performance than male U15 skiers, while there was no sex difference at the under 10-year (U10) level. In female U10 skiers, DBB_displacement_ was moderately associated with body height, while in all other subgroups, no confounding associations with anthropometrics or biological maturation were found. Biomechanically quantifying DBB performance may be considered a feasible and nonconfounded screening test approach in young skiers older than 6 years. Body height may represent a confounding bias in exclusively the U10 female skier cohort and, therefore, should be considered when interpreting the test results. In summary, this study provided sport-specific normative reference data that may be of equal interest to both researchers and sport practitioners.

## 1. Introduction

Competitive alpine skiing is known to be a sport with a strikingly high risk of injury, even at a young age [1,2]. The most frequently affected are the knee (e.g., ACL injuries and patellar tendon complaints) and the lower back (e.g., spinal disc degenerations) [1,2,3,4]. Core strength is a predominant critical factor for ACL injuries in youth skiers [5], and with respect to overuse-related spinal abnormalities, it has been shown that a smaller relative lumbar cross-sectional area of the multifidus muscle, as one of the stabilizing paraspinal muscles, was associated with a more frequent occurrence of disc protrusions and end plate changes in the spines of youth skiers [3].

Such findings are not surprising, since competitive alpine skiing places high demands on the physical fitness and musculoskeletal loading robustness of athletes [6,7]. Especially essential is the antirotation and rear-chain stabilization capacity of the skiers’ trunk, which is based on a well-coordinated activation of the deep lateral trunk, paraspinal, pelvic and posterior leg muscles. Superior stabilization capacity is needed to constantly remain in dynamic equilibrium while skiing despite perturbations due to constantly changing turn forces, ski-snow interactions and air drag, as well as impacts caused by bumps and gate contacts [8], and to counteract the ski-specific adverse loading patterns of the back, including frontal bending, lateral bending and torsion in the highly loaded spine, along with excessive exposure to adverse low-frequency vibrations [9,10]. Moreover, for preventing anterior cruciate ligament (ACL) injury inciting out-of-balance situations, as described in earlier studies [11,12], superior stabilization capacities may also be relevant.

**Figure 1:**
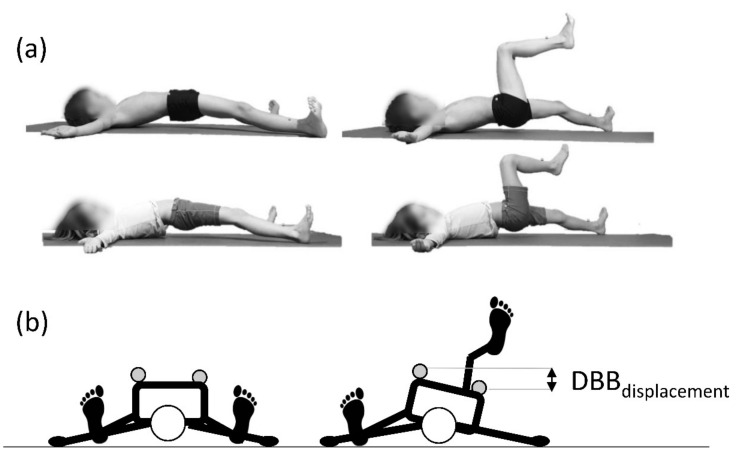
Lateral (**a**) and cranial (**b**) views of the deadbug bridging (DBB) exercise in (**left**) the initial position and (**right**) the maximum leg lift phase. Participants performed the leg lift sequence three times without the hip touching the ground. Markers are placed on clothing for the picture but are for actual testing placed on the skin.

Given these sport-specific demands, a biomechanical quantification of skiers’ stabilization performance during deadbug bridging (DBB) exercises, i.e., the assessment of the relative displacement of one of the anterior spina iliac superior markers with respect to the contralateral marker in the transverse plane (see Figure 1, Methods section), can be considered indicative of their global antirotation and rear-chain stabilization capacity. Indeed, a previous study found that biomechanically quantified DBB stabilization performance was related to both skiing performance and lower back overuse complaints in skiers of the under 16 years (U16) category [13]. Another study that examined the effects of an injury prevention program targeted to the specific injury patterns of youth skiers, which included DBB exercises, revealed a significantly lower prevalence of knee trauma and back overuse complaints [14].

While a biomechanical quantification of DBB performance and DBB exercises as preventative countermeasures have been thoroughly explored for skiers of the U16 category and the elite level in previous studies [13,14], to date, very little is known about the corresponding stabilization abilities in younger skiers, i.e., 6- to 15-year-old skiers. First, it is not a priori clear whether the coordinatively challenging motion task of DBB could be widely implemented for screening and training purposes in the youngest athletes. Second, sport-specific normative reference data for skiers of the youngest levels are lacking; however, they may be of equal interest to both researchers and sport practitioners. Third, while for U16 and elite skiers, the corresponding testing approach is an adequate method to quantify athletes’ antirotation and rear-chain stabilization capacity with no relevant confounders, knowledge about the dependency of DBB performance on age, sex and biological maturation in younger skiers is currently lacking.

Therefore, the primary aim of this study was to investigate the implementability of the DBB exercise in 6- to 15-year-old competitive alpine skiers and to determine their DBB performance as a function of age group and sex. The secondary aim was to investigate the associations of DBB performance with age, anthropometrics and maturity offset.

## 2. Materials and Methods

### 2.1. Study Design, Setting and Participants

This cross-sectional study builds upon the biomechanical data of 101 6- to 15-year-old male and female competitive alpine skiers (58 females and 43 males, Table 1). All data were collected as part of a physical fitness competition at the final event of the “SwissPass Smile Challenge” during the 2021 off-season period in Switzerland and after the event reanalysed for the purpose of the current study. From a total of 260 youth skiers who took part in the final event in groups of five, two team members were randomly selected to perform the DBB exercise-based competition discipline. The inclusion criterion was participation in the corresponding competition discipline, while there were no exclusions. Based on their age and sex, participants were pooled into one of the four groups: U10 female: ≤10-year-old girls, U10 male: ≤10-year-old boys, U15 female: 11- to 15-year-old girls, and U15 male: 11- to 15-year-old boys (Table 1). The reuse of the anonymized dataset was approved by the Cantonal Ethics KEK Zürich (KEK-ZH-NR: 2021-01044). Patient consent was waived because the present study used anonymized data, and the underlying study protocol was therefore judged by the Cantonal Ethics Committee KEK Zurich as not falling under the scope of the Human Research Act (HRA).

### 2.2. Data Collection and Evaluation

During the above-described physical fitness competition, one discipline was the biomechanical quantification of DBB performance. All participants underwent (1) baseline assessments for determining age, anthropometrics and biological maturation, (2) final instructions regarding DBB exercise, (3) marker attachment, and (4) data collection.

Prior to the event, a video tutorial was made available for all participants to practice the DBB exercise beforehand and ensure that all participants were sufficiently familiarized with the exercise (https://ars.els-cdn.com/content/image/1-s2.0-S1466853X20304168-mmc1.mp4, accessed 18 February 2022) [13].

#### 2.2.1. Age, Anthropometric Data and Determination of Biological Maturity

Prior to testing, chronological age, body weight (0.5 kg, Seca, Hamburg Germany), body height, and sitting height (1 cm, determined by measuring tape) were determined. Maturity offset was calculated according to Mirwald et al. [15], who proposed a noninvasive methodology that uses empirically developed sex-specific formulas to calculate maturity offset (MO) based on anthropometric data and chronological age. MO represents a point in time before or after skiers reach their age at peak height velocity (APHV). APHV is obtained by subtracting the MO from the chronological age. A negative MO indicates the time when APHV will be reached, whereas a positive MO indicates that the APHV has already been passed. In accordance with Kiers et al. [16], MO and the corresponding APHV were only calculated for the group of U15 skiers, assuming that the Mirwald et al. [15] equation has limited validity for the U10 group [17,18,19].

#### 2.2.2. DBB Performance Assessment

DBB performance was determined biomechanically while the skiers performed the DBB exercise. DBB exercise is a low dynamic closed-chain stabilization exercise addressing typical components of mechanisms leading to back overuse injuries in alpine ski racing [10,13]. The DBB exercise was instructed to be executed as follows: In the starting position, the participant lays on his/her back with the arms positioned 90° to the side (open hands pointing upwards) and the lower limbs extended at the knee and abducted at the hip (Figure 1a, left). This approximately corresponded to a position where the ankle and elbow were at the same lateral distance from the centre of the body. The toes are actively pulled into dorsiflexion. Now the participants lift their pelvis approximately one fist width high. The test task was to stabilize the pelvis while lifting one leg off the ground and moving the knee toward the chest until the thigh was perpendicular to the ground (Figure 1a, right). The position was held for 3 s, followed by controlled positioning of the heel on the ground (Figure 1a, left). This is repeated three times without the hip touching the ground. During exercise execution, the following cues were verbally provided: “pelvis up”; “left/right knee to the towards the chest”; “and back”; “controlled leg drop”; “and “pelvis down”.

To biomechanically quantify DDB performance, reflective markers were attached at the skin above the right and left anterior spina iliaca superior (ASIS) and the left and right tibia. The movement of the reflective markers was collected using a 3D motion capture system (Vicon, Oxford, UK) with 8 infrared cameras (Vero 1.3) set at 200 Hz and 1 synchronized DV camera (Vue) set at 50 Hz. The parameter of interest was the maximal vertical difference between the left and right ASIS markers during the leg lift phase (Figure 1b). The leg lift phase was determined using the vertical movement of the tibia marker with a relative threshold of 50 mm vertical displacement to the lowest tibia marker position. Foot-off was determined as −25 frames (−125 ms) before reaching the threshold during the upwards movement of the limbs. Foot strike was defined as +50 frames (+250 ms) after the threshold was reached when the limb moved downwards. The mean value of the right and left exercise execution of the three performed cycles was calculated and used as the global parameter, called DBB_displacement_. Such an approach to determine DBB_displacement_ has been shown to be reliable (ICC(3,1) and 95% CI of 0.81 [0.61, 0.93]; within-subject standard error of measurement (SEM) was 3.89 mm [3.16 mm, 5.12 mm]) and clinically meaningful (i.e., associated with back overuse complaints; an increase of DBB_displacement_ by 1 mm increased the relative probability of suffering from substantial back overuse by 4.9%) in a previous study with U16 male and female skiers [13].

### 2.3. Statistical Analysis

Statistical analysis was performed using IBM SPSS software (Version 26) and assuming a significance level of *p* < 0.05. Normal distribution of data was checked using the Shapiro–Wilk test, graphical techniques and shape parameters (skewness and kurtosis coefficients), as suggested by Razali and Wah [20]. In cases where the Shapiro–Wilk test revealed significant results but corresponding skewness and kurtosis values were markedly below common reference boundaries of substantial departure from normality (<2.0 and <7.0 as defined by West et al. [21]), standard parametric tests were backed-up by bias corrected accelerated (BCa) bootstrapping with 10,000 samples. Bootstrapping was applied for the testing of the following parameters and subgroups: age (U10 female skiers, U15 female skiers); DBB_displacement_ (U10 male skiers); and maturity offset (U15 female skiers, U15 male skiers).

All anthropometric data were evaluated with respect to the allocated age group (U10 vs. U15) and sex (female vs. male) differences by the use of a multivariate analysis of variance (MANOVA) with Bonferroni corrections of pairwise comparison. Biological maturation and APHV were evaluated using independent sample *t* tests. Age (U10 vs. U15) and sex (female vs. male) differences in DBB_displacement_ values were analysed by two-way analysis of variance (ANOVA), with Bonferroni correction for post hoc tests. Pairwise comparisons were additionally illustrated by mean and 95% CI plots. Correlations of DBB_displacement_ with the potential confounders age, anthropometrics, maturity offset and APHV were tested using Pearson’s correlation coefficient (r) and the coefficient of determination (R^2^).

## 3. Results

### 3.1. Baseline Characteristics and Biological Maturation

The baseline characteristics and biological maturation of the four investigated subgroups are presented in Table 1. On a multivariate level, a significant age effect (U10 vs. U15) existed in the baseline parameters: age (*p* ≤ 0.001, η^2^ = 0.720), body height (*p* ≤ 0.001, η^2^ = 0.577) and body weight (*p* ≤ 0.001, η^2^ = 0.429). In more detail, post hoc analysis revealed differences between the U10 and U15 groups in age (*p*-value: male and female *p* ≤ 0.001; Cohen’s d: female d = 17.09 and male d = 20.07), body height (*p*-value: female and male *p* ≤ 0.001; Cohen’s d: female d = 14.64 and male d = 9.49) and body weight (*p*-value: female and male *p* ≤ 0.001; Cohen’s d: female d = 11.84, and male: d = 5.01). The only sex difference occurred in the U15 group for maturity offset (*p* = 0.001, d = 6.09) and APHV (*p* < 0.001, d = 10.08). The U15 girls were with an average MO of 1.3 ± 1.3 years slightly past their APHV, while the U15 boys had with a MO of −0.6 ± 1.5 years just or just not yet reached their APHV; hence, their peak growth spurt is yet to come. No interaction effect between sex and age group was detected in any of the analysed parameters.

### 3.2. Differences in Absolute DBB_displacement_ with Respect to Age and Sex

First, it is important to note that despite their young age, all participants were able to perform the DBB exercise to an “adequate” quality level (i.e., executing the DBB exercise as instructed, with the pelvis not touching the floor during the leg lift). The resulting reference data separated for each age and sex group are presented in Table 2.

**Figure 2:**
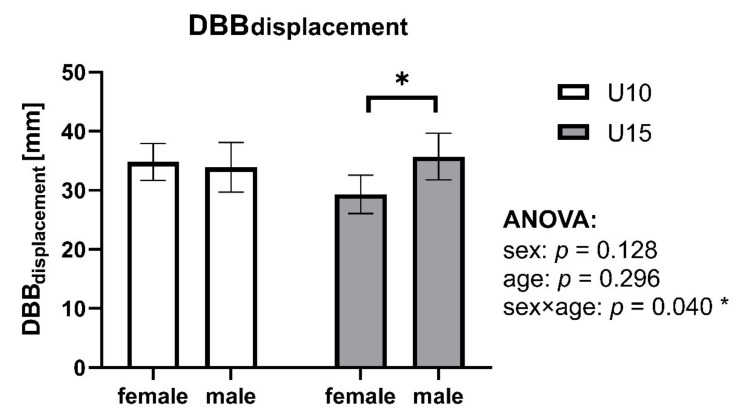
DBB_displacement_ separated by age and sex. Data are expressed as the mean ± 95% CI. Level of significance based on a two-way ANOVA: * sign (*p* < 0.05).

Univariate ANOVA revealed no significant main effect for age and sex but a significant interaction effect of sex×age (*p* = 0.040, η^2^ = 0.043) (Figure 2). While male and female skiers of the U10 group showed comparable results (*p* = 0.705, d = 0.10), a significant difference was found between sexes in the U15 group (*p* = 0.013, d = 0.49). Female U15 skiers showed with a mean DBB_displacement_ difference of −6.3 mm (−11.3 to −1.4 lower and upper boundary of 95% CI) better DBB performance than their male counterparts.

### 3.3. Association between DBB_displacement_, Age, Anthropometrics, Maturity Offset and APHV

In Table 3, correlations (including r, R^2^ and p values) between DBB_displacement_ and age, body height, body weight, maturity offset and APHV are presented. The only significant positive correlation was observed in the U10 female group between DBB_displacement_ and body height (r = 0.413, R^2^ = 0.170, *p =* 0.021).

## 4. Discussion

The major findings of the current study were as follows: (1) despite their young age, all participants were able to correctly perform the DBB exercise after a short introduction and exercise phase; (2) DBB_displacement_ did not significantly differ between female and male U10 skiers, while in the U15 group, female skiers presented a better DBB performance than male skiers; (3) in female U10 skiers, there were significant associations between DBB_displacement_ and body height (the taller the greater the DBB_displacement_), while among male U10 and female and male U15 skiers, no significant associations were observed in any of the potential confounders analysed.

### 4.1. Implementability of DBB Exercises in Competitive Alpine Skiers Aged 6- to 15-Years

First, it should be emphasized that according to our observations as part of this study, the DBB exercise can already be used with 6- to 15-year-old skiers. All participants were able to correctly perform the DBB bridging exercise (i.e., executing it as instructed, with the pelvis not touching the floor during the leg lift) after a short introduction and practice phase, which is also reflected in an acceptable variation of the test results. All test results were within the range of 37 mm DBB_displacement_. Furthermore, the interquartile ranges (Q_1_–Q_3_) observed in the current study for U10 skiers were with <16 mm DBB_displacment_ of comparable magnitude to the one observed for U16 (<17 mm) and elite skiers (<15 mm) in an earlier investigation [13]. This means that the relative displacement of one of the anterior spina iliac superior markers with respect to the contralateral marker in the transverse plane was relatively small on average, with similar variation compared with professional athletes, indicating an acceptable antirotation and rear-chain-stabilization capacity of all 6- to 15-year-old skiers. Thus, despite their young age, all participants seemed to possess the muscular strength capacity and the coordinative skills to perform this exercise.

### 4.2. Differences in Baseline Characteristics and DBB_displacement_ with Respect to Age Group and Sex

Within the U10 group, no significant baseline characteristic differences between male and female skiers were observed, while in the U15 group, there were significant sex differences with respect to maturity offset. Despite their similar chronological age (13.6 ± 1.3 years female skiers, 13.3 ± 1.4 years male skiers), female skiers had already passed their APHV by on average 1.3 ± 1.3 years, while the male skiers were on average −0.6 ± 1.5 years before reaching APHV. As such, our values conform with reported APHV values among European athletes of different sports, which vary between 12.0 to 13.2 years in girls and 12.9 to 15 years in boys [22].

Given the considerable biological maturation differences between female and male U15 skiers, the better DBB performance of U15 females observed in this study is entirely plausible. Maturation-related differences are most evident during the pubertal transition from early to mid-adolescence (13 to 15 years) [22]. However, as adolescence further progresses, i.e., between 16 and 18 years, the maturation-related differences decrease and largely disappear in nonathletes and athletes [22]. This was also observed in relation to DBB performance in elite skiers, where gender differences were no longer present after puberty, i.e., when both females and males maturing on average 1.5 years later had passed their APHV [13].

With respect to antirotation and rear-chain stabilization, i.e., DBB performance, the multifidus muscle plays an important role as a muscle belonging to the paraspinal back muscles [23]. Of interest in a similar context is the linear relationship between the relative multifidus cross-sectional area and skier maturity offset, which was shown in a previous study to suggest that skiers closer to their APHV have less developed paraspinal muscles and thus a limited ability to stabilize the spine [3]. Although speculative and likely only one aspect in a multifactorial setting, this relationship may be evident in the DBB_displacement_ shift in the vulnerable ages of the U15 skiers of this study and U16 skiers in a previous study [13].

### 4.3. Association of DBB_displacement_ with Age, Anthropometrics and Maturity Offset in U10 and U15 Skiers

In female U10 skiers, DBB_displacement_ was moderately associated with body height, while in all other subgroups, no confounding associations with anthropometrics or biological maturation were found. Accordingly, it seems that in the U10 group, anthropometric aspects may play a confounding role in DBB performance, which is why the interpretation of the DBB data in this subgroup has to be made with caution. In this context, normalization to body height (DBB_displacement_ divided by body height) eliminated the positive association in the U10 female cohort (r = 0.267, R^2^ = 0.07, *p =* 0.147). Such normalization may be one reasonable approach to address this issue.

### 4.4. DBB Performance in 6- to 15-Year-Old Skiers—Why It May Matter

In competitive alpine skiing, a superior antirotation and rear-chain stabilization capacity plays—independent of age—an important role in the prevention of both lower back overuse injuries [13] and traumatic injuries, such as ACL ruptures [11,12]. Thus, implementing specific trunk stabilization training at young ages with accompanying screening tests could detect impairments in early stages. Although this paper cannot provide any information about direct relationships to occurring injuries, assessing the feasibility, presenting reference data and investigating potential confounders in the group of 6- to 15-year-old skiers is a first crucial step toward effective injury prevention.

In addition, it should be highlighted that lower back pain is already a frequent complaint at the youth level [2,24,25] and occurs along with high rates of overuse-related structural abnormalities in the lumbar spine of youth competitive alpine skiers [3,26]. As a potential biomechanical mechanism of back overuse injuries in alpine ski racing, a combination of (a) heavy mechanical loads acting on the spinal structures, which accumulate over the entire training season, (b) typical loading patterns while skiing, which include an unfavourable occurrence of frontal bending, lateral bending and torsion in the highly loaded spine and (c) excessive exposure to adverse low-frequency vibrations during ski racing, might explain the high injury numbers [9,10]. In all three aspects, the structural morphology of the paraspinal muscles and their functional performance for stabilizing the trunk play a crucial role [27], as such a link between the cross-sectional area of the multifidus muscle and overuse injuries in U16 skiers has already been shown in a previous study [3]. Moreover, an increase of 1 mm in pelvic drops during DBB performance (i.e., DBB_displacment_) increased the relative probability of suffering from substantial back overuse injury in U16 skiers by 4.9% [13]. However, although this evidence is theoretically plausible for younger skiers aged 6 to 15 years, it still needs to be confirmed by further studies.

With regard to traumatic injuries, such as ACL ruptures, superior trunk stabilization capacities are crucial to constantly remain in dynamic equilibrium while skiing despite perturbations. Accordingly, such skills may help to avoid out-of-balance situations that, as described in previous studies [11,12], typically lead to ACL injuries.

### 4.5. Limitations

The following potential limitations need to be considered when interpreting the study findings.

First, in the current study, antirotation and rear-chain stabilization capacity was quantified by a single global kinematic parameter called DBB_displacement_, while the underlying single muscle control mechanisms and aspects of muscle interplay remain uninvestigated. However, despite DBB being a standardized exercise, it addressed the entire lateral and rear-chain, meeting the demands of direction-specific and synergistic coactivation of global and local trunk, hip and leg muscles [28] including both static and slow movements [29].

Second, the cross-sectional design describing DBB performance in two sexes and two age groups (U15 and U10) is limited to fully picture skiers’ entire development process with respect to DBB performance across their sportive careers. However, together with an earlier study by Ellenberger et al. [13], reference data are now available for U10, U15, U16 and elite skiers.

Third, to determine APHV, the valid prediction equations by Mirwald et al. were chosen [19]. Despite the determination of APHV via X-rays of the wrist representing a more accurate approach [30], the current method allowed an easy implementable determination without the need for radiation [19] and extra cost under in-field conditions.

Fourth, a reliability analysis of DBB testing in the specific 6- to 15-year-old skier cohort of this study was not feasible due to the competitive nature of the final event of the “SwissPass Smile Challenge” at which data were collected. However, a previous study showed that the test-retest reliability of the proposed biomechanical approach for DBB quantification in healthy adults is *good* and that the (standardized) within-subject SEMs are *moderate*. Since there are no known confounding effects of age, maturity, or anthropometry in DBB testing in previous studies, it is reasonable to assume that these reliability values are also similar for the cohort of this study. The only exception might be the subgroup of female U10 skiers, for whom (due to certain confounding relationships with height) test-retest reliability might be somewhat different.

## 5. Conclusions

The DBB exercise is a holistic approach to quantify skiers’ antirotation and rear-chain stabilization capacity for intervention and screening purposes. DBB screening might provide important feedback on the antirotation and rear-chain stabilization capacity of the skiers’ trunk, which is based on a well-coordinated activation of the deep lateral trunk, paraspinal, pelvic and posterior leg muscles, could detect possible deficits. Early recognition and effective intervention may reduce the risk of overuse injury or its severity, and consequently prevent the absence of training and competition in the follow-up. This study underlines the implementability of DBB for screening purposes in skiers aged 6- to 15-years and provides for the first-time reference data for a skiing-specific cohort of youth athletes of both sexes in the same age group. Since confounding factors were found in the group of U10 female skiers, in this specific cohort, DBB data need to be interpreted with caution.

## Figures and Tables

**Table 1:** Baseline characteristics.

	U10		U15		*p* Values		
Variables	Female	Male	Female	Male	Sex	Age	Sex × Age
sample size	31	19	27	24			
age [years]	9.8 ± 0.9 (6.7–10.8)	9.5 ± 0.9 (7.9–10.8)	13.6 ± 1.5 * (11.1–15.6)	13.3 ± 1.4 * (11.2–15.6)	0.172	<0.001 *	0.947
body height [cm]	137.7 ± 6.8 (121.0–148.5)	139.7 ± 7.5 (129.0–154.0)	159.5 ± 8.5 * (143.0–172.5)	161 ± 13.3 * (140.0–180.5)	0.365	<0.001 *	0.904
body weight [kg]	31.3 ± 6.0 (20.0–49.0)	33.4 ± 8.4 (25.0–54.0)	49.2 ± 9.8 * (31.0–64.0)	49.5 ± 14.1 * (28.0–74.0)	0.532	<0.001 *	0.669
maturity offset [years]	---	---	1.3 ± 1.3 (−1.1–2.8)	−0.6 ± 1.5 ^#^ (−2.7–1.6)	<0.001 ^#^	---	---
APHV [years]	---	---	12.4 ± 0.4 (11.6–13.0)	13.9 ± 0.7 ^#^ (12.4–15.6)	<0.001 ^#^	---	---

Data are expressed as the mean ± SD (min-max). Significant differences identified by post hoc tests: ^#^ = significant difference between sexes within the same age group; * = significant difference between age groups within sexes. The presented *p* values for age, body height, and body weight are based on MANOVA. Main effects: ^#^ sex (female vs. male), * age group (U10 vs. U15). Interaction effect: +(sex × age). The presented *p* values for maturity offset and APHV (data exist for U15 only) are based on independent sample *t* tests.

**Table 2:** DBB_displacement_ reference data for female and male U10 and U15 competitive alpine skiers.

DBB_displacement_ [mm]	Mean	SD	Min	Q1	Median	Q3	Max
U10 female	34.8	8.5	21.1	26.6	35.4	42.4	48.4
U10 male	33.9	8.7	26.0	27.4	31.1	38.1	58.5
U15 female	29.3	8.2	12.8	22.6	28.4	33.9	44.6
U15 male	35.7	9.3	20.0	28.1	34.8	43.1	56.5

**Table 3:** Pearson’s correlations coefficient (r), determination coefficient (R^2^) and *p*-values for the correlation between DBB_displacement_ and age, body height, body weight, APHV and maturity offset.

		DBB_displacement_ [mm]			
		U10		U15	
Variables		Female	Male	Female	Male
age [years]	r (R^2^)	−0.141 (0.020)	−0.010 (<0.001)	−0.178 (0.032)	0.030 (0.001)
	*p*-value	0.451	0.967	0.373	0.889
body height [cm]	r (R^2^)	0.413 (0.171)	0.128 (0.016)	−0.152 (0.023)	−0.034 (0.001)
	*p*-value	0.021 *	0.602	0.449	0.875
body weight [kg]	r (R^2^)	0.295 (0.087)	0.377 (0.142)	−0.295 (0.087)	0.078 (0.006)
	*p*-value	0.108	0.111	0.108	0.716
maturity offset [years]	r (R^2^)	---	---	−0.195 (0.038)	−0.013 (0)
	*p*-value	---	---	0.330	0.952
APHV [years]	r (R^2^)	---	---	−0.028 (0.001)	0.087 (0.007)
	*p*-value	---	---	0.888	0.688

For maturity offset and APHV data exist for U15 only. Level of significance: * *p* < 0.05.

## Data Availability

Restrictions apply to the availability of these data. Data were obtained from Swiss-Ski and are available from the authors with the permission of Swiss-Ski.
